# Discovery of a putative blood-based protein signature associated with response to ALK tyrosine kinase inhibition

**DOI:** 10.1186/s12014-020-9269-6

**Published:** 2020-02-07

**Authors:** Mathilde Couëtoux du Tertre, Maud Marques, Suzan McNamara, Karen Gambaro, Cyrla Hoffert, Lise Tremblay, Nicole Bouchard, Razvan Diaconescu, Normand Blais, Christian Couture, Vincent Pelsser, Hangjun Wang, Laura McIntosh, Valérie Hindie, Stephane Parent, Laetitia Cortes, Yannick-André Breton, Gwenael Pottiez, Pascal Croteau, Valerie Higenell, Luisa Izzi, Alan Spatz, Victor Cohen, Gerald Batist, Jason Agulnik

**Affiliations:** 1 Segal Cancer Centre, Jewish General Hospital, McGill University, Jewish General Hospital, 3755, Chemin Cote Ste-Catherine, Montreal, QC H3T1E2 Canada; 2 Exactis Innovation, Montréal, QC Canada; 3 Institut universitaire de cardiologie et pneumologie de Québec, Université de Laval, Québec, QC Canada; 4 Centre hospitalier universitaire de Sherbrooke, Sherbrooke, QC Canada; 5 Hopital Sacré-Cœur Montréal, Montréal, QC Canada; 6 Centre hospitalier universitaire de Montréal, Montréal, QC Canada; 7 Caprion, Montreal, QC Canada

**Keywords:** Protein signature, Crizotinib, Liquid biopsy, NSCLC, Prediction of response

## Abstract

**Background:**

ALK tyrosine kinase inhibition has become a mainstay in the clinical management of ALK fusion positive NSCLC patients. Although ALK mutations can reliably predict the likelihood of response to ALK tyrosine kinase inhibitors (TKIs) such as crizotinib, they cannot reliably predict response duration or intrinsic/extrinsic therapeutic resistance. To further refine the application of personalized medicine in this indication, this study aimed to identify prognostic proteomic biomarkers in ALK fusion positive NSCLC patients to crizotinib.

**Methods:**

Twenty-four patients with advanced NSCLC harboring ALK fusion were administered crizotinib in a phase IV trial which included blood sampling prior to treatment. Targeted proteomics of 327 proteins using MRM-MS was used to measure plasma levels at baseline (including pre-treatment and early treatment blood samples) and assess potential clinical association.

**Results:**

Patients were categorized by duration of response: long-term responders [PFS ≥ 24 months (n = 7)], normal responders [3 < PFS < 24 months (n = 10)] and poor responders [PFS ≤ 3 months (n = 5)]. Several proteins were identified as differentially expressed between long-term responders and poor responders, including DPP4, KIT and LUM. Next, using machine learning algorithms, we evaluated the classification potential of 40 proteins. Finally, by integrating the different analytic methods, we selected 22 proteins as potential candidates for a blood-based prognostic signature of response to crizotinib in NSCLC patients harboring ALK fusion.

**Conclusion:**

In conjunction with ALK mutation, the expression of this proteomic signature may represent a liquid biopsy-based marker of long-term response to crizotinib in NSCLC. Expanding the utility of prognostic biomarkers of response duration could influence choice of therapy, therapeutic sequencing, and potentially the need for alternative or combination therapy.

*Trial registration* ClinicalTrials.gov, NCT02041468. Registered 22 January 2014, https://clinicaltrials.gov/ct2/show/NCT02041468?term=NCT02041468&rank=1

## Background

Non-small cell lung cancer (NSCLC) represents 85% of lung cancers, 64% of which contain oncogenic driver mutations [1, 2]. In 3–7% of the cases, rearrangements in the anaplastic lymphoma kinase (ALK) gene is observed and is demographically associated with younger patients who are light- or non-smokers [3, 4]. Its main fusion partner is echinoderm microtubule-associated protein-like 4 (EML4) found in about 80% of the patients, with more than a dozen different EML4-ALK variants documented [5]. The remaining 20% is composed of low frequency fusions between ALK and numerous other genes like KIF5B and TFG [6].

The identification of ALK fusion as the main driver in this subset of NSCLC, led to the pharmacological development of drugs inhibiting ALK kinase activity. Crizotinib was the first molecule to be FDA approved and was used as first line therapy in ALK fusion positive NSCLC patients with a 74% response rate. Unfortunately, most patients progress within 1–2 years due to acquired resistance occurring via two types of mechanisms: on-target with the acquisition of secondary mutations in the tyrosine kinase domain of ALK, decreasing drug efficacy and, off-target through the activation of alternative signaling pathways. Over the last few years, second- and third-generation ALK inhibitors have been developed to overcome some of the resistance mechanisms link to crizotinib exposure, as well as, increased potency, selectivity and blood–brain barrier permeability [7–11]. Resistance to next-generation ALK-TKIs also arises or develops and is more difficult to overcome with numerous patients carrying compound mutations in ALK or developing/activating off-target mechanisms. The perpetual adaptation of tumor cells to ALK-TKIs leading to acquired resistance remains a major challenge in treating ALK fusion positive NSCLC patients, and identification of prognostic biomarkers could help guide treatment choice, as well as sequence of administration.

This study aimed to evaluate prognostic proteomic biomarkers predictive of response to crizotinib in patients diagnosed with locally advanced or metastatic ALK fusion positive NSCLC. Patients were administered crizotinib according to standard of care, then categorized into three groups by duration of response. Baseline blood samples were analyzed by multiple-reaction monitoring-mass spectrometry to identify plasma protein levels in patients prior to therapy. We identified several proteins significantly differentially expressed in long-term responders compared to poor responders. In parallel, using machine-learning algorithms, we identified 40 proteins more likely to predict patient duration of response and propose that 22 of these proteins should be investigated further to refine a molecular signature of long-term response to crizotinib.

## Methods

### Study oversight

We conducted a prospective observational study (NCT02041468) at 5 major cancer centres in Canada. The study was approved by the institutional review board at each participating hospital. All patients provided written informed consent prior to any study specific procedures.

### Trial design, treatment and assessments

This phase IV study was performed in a real-world context for locally advanced or metastatic ALK fusion positive NSCLC patients between January 31, 2014 and July 31, 2018 (cut-off date).

ALK rearrangement status was assessed on FFPE primary lung tumor or fine needle aspirates either by immunohistochemistry using ALK antibody clones 5A4 (Novocastra or Biocare) or D5F3 (Cell Signalling Technologies), or by fluorescent in situ hybridization (FISH) using the Vysis LSI ALK Break Apart FISH Probe Kit.

Study objectives included confirmation of measures of efficacy of crizotinib therapy (progression-free survival [PFS], disease control rate [DCR] and time to treatment discontinuation) and assessment of blood-based biomarkers of response or resistance to crizotinib. Response to treatment was assessed by radiological imaging within 30 days of starting treatment and every 8–12 weeks during treatment until progression. Objective response was measured at each evaluation using the Response Evaluation Criteria in Solid Tumors (RECIST) v.1.1 [12].

Treatment with crizotinib followed standard of care. Patients received oral crizotinib at a dose of 250 mg twice daily, or 200 mg twice daily in the case of toxicity and continuation beyond progression of disease was left to the opinion of the treating physician. Five out of 24 patients (20.8%) were already receiving crizotinib therapy when enrolled in this study. Two out of these 5 patients were treated with crizotinib in combination with the HSP90 inhibitor onalespib (AT13387; Astex Pharmaceuticals) in a previous clinical trial (NCT01712217). A total of 22 samples were analyzed for individual protein expression levels and combined protein panels.

### Blood sample collection

Prior to treatment administration, blood samples were drawn, collected in k_2_EDTA Vacutainer^®^ tubes and centrifuged within 60 min of collection at 1500*g* for 15 min at room temperature. Plasma was harvested, aliquoted and stored at − 80 °C.

### Target and peptide selection assay

Using an untargeted mass-spectrometry approach, more than four thousand proteins were identified in tissue samples from patients with both ALK-fusion positive and -fusion negative NSCLC not part of the patients described in the present study. Three hundred twenty-seven (327) target proteins represented by 900 peptides were selected from the discovery study and optimization phase, which included a large fraction of secreted proteins and additional targets of interest.

### Blood sample processing and multiple reaction monitoring (MRM) analysis

Samples (30 μL) were depleted of high and medium abundance proteins by immunoaffinity chromatography using commercially available IgY14-SuperMix resin (10 × 100 mm column, Agilent) and a 1200 HPLC instrument (Agilent) equipped with a thermostated autosampler and fraction collector.

The unbound fraction [flow through (FT)], containing the remaining lower abundance proteins, was collected for each sample and freeze-dried prior to digestion. The FT fractions were re-solubilized and digested with trypsin [1:10 (w:w) enzyme: protein ratio, Promega Corporation] at 37 °C with shaking overnight. The digested samples were spiked with 20 μL of a 20 pmol/mL crude stable isotope-labeled (SIL) peptide mix (see section below) and desalted using Oasis mixed-mode cation-exchange (MCX) resin in a 96-well plate format (Waters). Desalted peptides were vacuum evaporated and stored at − 20 °C until MRM analysis.

For MRM analysis, the samples were re-solubilized and spiked with 5 internal standard peptides for instrument monitoring. Ten μg of each sample was injected onto a NanoAcquity UPLC (Waters) coupled to a QTRAP 5500 mass spectrometer. Peptide separation was achieved using a Halo Peptide ES-C18 500 μm × 10 cm column, 2.7 μm particle size (Advanced Materials Technology). The gradient time was 30 min, and the flow rate was 18 µL/min. Peptide signals were integrated using MultiQuant software (AB Sciex). The CE value giving the most intense signal for each transition was determined using in-house software developed by Caprion.

### Differential protein expression analysis

To generate a protein signature predictive of a long-term response in ALK fusion positive NSCLC, protein abundance ratios from long-term responders and normal responders were compared. To be included in the signature, proteins needed to be differentially expressed between long-term and normal responders with a P-value < 0.1 and have a similar fold change sign in long-term versus poor responder groups comparison, resulting in 15 proteins being selected.

The proteomic expression matrix containing the abundance of the 126 proteins detected was used as input to perform hierarchical clustering (Euclidean, complete linkage) of the protein signature, as well as principal component analysis. The ssGSEA Projection tool (https://genepattern.broadinstitute.org) was used on the same matrix as the protein signature, and the score obtained for each patient was visualized. This analysis leverages the presence of multiple, correlated sources of information about biological processes (the proteomic expression matrix) to determine the activity level of underlying biological processes that belie coordinated expression patterns of particular genes or proteins (the signature). The purpose of this multivariate analysis was to identify potential biomarkers that interacted with each other that would not have been detected in simple univariate analyses.

### Panel analysis strategy

To focus the search of biomarker candidates that could act in concert to predict response duration, four algorithms (extreme gradient-boosted decision trees, least absolute shrinkage and selection, ridge regression, and elastic net regression [13–17]) were given centered, unit variance protein intensities and trained to discriminate long-responders from either non-responders or normal responders. Classifications were repeated with 100 randomized column orders with 100 cross-validations each, using half of the data as a training set. Estimates for each algorithm were subsequently averaged across these 10,000 trials. An importance measure was then calculated (gain for decision trees or squared coefficient for penalized regressions) for each protein. Following a rate-change detection scheme (previously described [18]) performed on the survivor function of importance values attributed by each algorithm, changes in importance survival rate were tested. All proteins with importance greater than that which marked a change in survival rate were considered selected by an algorithm. Proteins were considered for panel analyses if at least one algorithm had selected them in either classification. A resulting 52 proteins were selected for panel testing.

Panels of every combination of up to 3 of these selected proteins were fit by bias-reduced general linear modeling (R package *brglm*). The performance of each panel was assessed by calculating the area under the receiver operating characteristic (ROC) curve (AUC). This was estimated 100 times for each panel using stratified sampling to split the data into halves that served as training and test sets. Logistic regression models were fit to the training half and used to calculate out-of-sample predictions for the test half and the AUC of the resulting cross-validation sample. For each cross-validation sample, a null hypothesis AUC was empirically determined by fitting logistic regression models to 50 shuffles of the training set labels with respect to their protein levels and taking the median out-of-sample AUC. The difference between this null AUC and the theoretical null of 0.5 (the “optimism”) was then removed from the cross-validation sample’s AUC.

Overall, optimism-corrected performance for a panel was calculated as the median across those 100 estimates; confidence intervals were derived from the 2.5 to 97.5% quantiles. ROC curves were generated using the same cross-validation procedure, averaging sensitivity and specificity values across all 100 cross-validation trials. To assess protein contributions to panels, the proportion of panels with optimism-corrected AUC greater than 0.85 containing the protein was calculated.

## Results

### Patient characteristics and clinical outcome

**Fig. 1:**
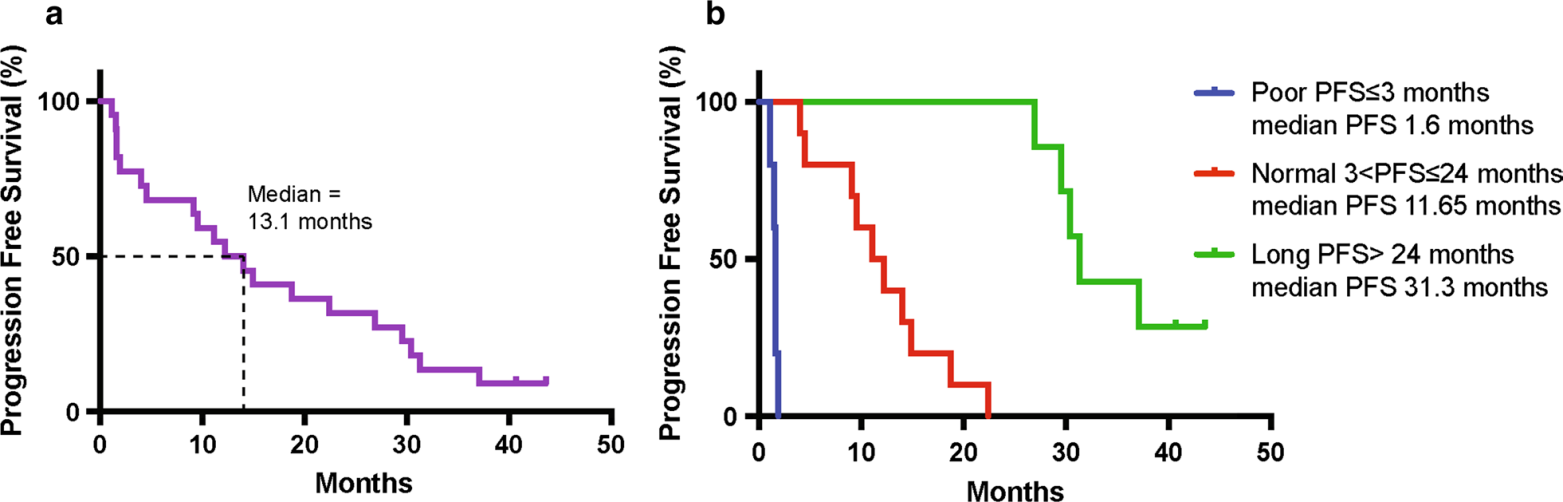
Progression-free survival. **a** Progression-free survival for the entire cohort. **b** Cohort stratified by duration of response

Twenty-four ALK fusion positive NSCLC patients were enrolled and administered crizotinib, with the aim of identifying prognostic proteomic and genomic biomarkers of response to crizotinib [19]. This study showed that there are likely multiple prognostic genomic biomarkers besides ALK mutations, that could be reflected in proteins other than ALK fusion-related protein products. The median PFS was 13.1 months (range 1.1–43.6 months, 95% CI 4–26.9 months, Fig. 1a), 2.2 months longer than reported in the literature (10.9 months) [20]. Interestingly, we observed a subset of patients with a durable response to crizotinib (≥ 24 months), which was driving this overall increase in PFS. Following this observation, patients were categorized into 3 groups based on PFS (Fig. 1b and Additional file 1: Table S1): poor, normal and long-term responders. Poor responders (5 patients) exhibited disease progression at first radiologic disease evaluation following treatment initiation (PFS ≤ 3 months). Normal responders (10 patients) experienced stable disease or an initial response but progressed after 3 to 24 months (3 < PFS ≤ 24 months). The upper cut-off for normal responders was established based on published data showing the duration of response to ALK-TKIs typically lasts no longer than 2 years. Long-term responders (7 patients) demonstrated a PFS greater than 24 months (PFS > 24 months). PFS was unknown for one patient withdrawn due to toxicity, and for one patient who withdrew consent.

### Selection of proteins for the multi-reaction monitoring analysis

A previous untargeted mass-spectrometry study using tissue samples from an independent patients cohort with both ALK-fusion positive and fusion negative NSCLC led to the identification of 327 proteins represented by 900 peptides, which include a large fraction of secreted proteins and additional targets of interest for this type of cancer [21]. In this study, using the aforementioned proteins, a targeted proteomic approach was performed on pre-treatment plasma samples to identify biomarkers predicting duration of response. From the initial set of 327 proteins monitored by the targeted approach, measurements were obtained for 126 proteins across most samples. Two complementary methods were used to discover proteins with prognostic potential in the present cohort: differential expression and classifier analysis.

### Differential expression analysis

First, we investigated the presence of signal in our data by identifying proteins differentially expressed in the pre-treatment or early treatment blood samples between the different groups of patients. The abundance ratio for each protein was compared between long-term and normal responders or long-term and poor responders (Additional file 2: Fig. S1a). A one-way ANOVA of the effect of patient group on expression levels was performed; multiple comparison corrections (q-values) were computed according to Benjamini & Hochberg [22]. We were particularly interested in the long-term versus normal comparison, but unsurprisingly, considering the small sample size, no protein reached significance after correction for false discovery rate. As a result, we ranked these proteins according to their p-value and selected the top 15 proteins (Additional file 3: Table S2) with a difference in abundance between long-term and normal groups (p < 0.1), which were also differentially expressed in the same direction between long-term and poor responders.

**Fig. 2:**
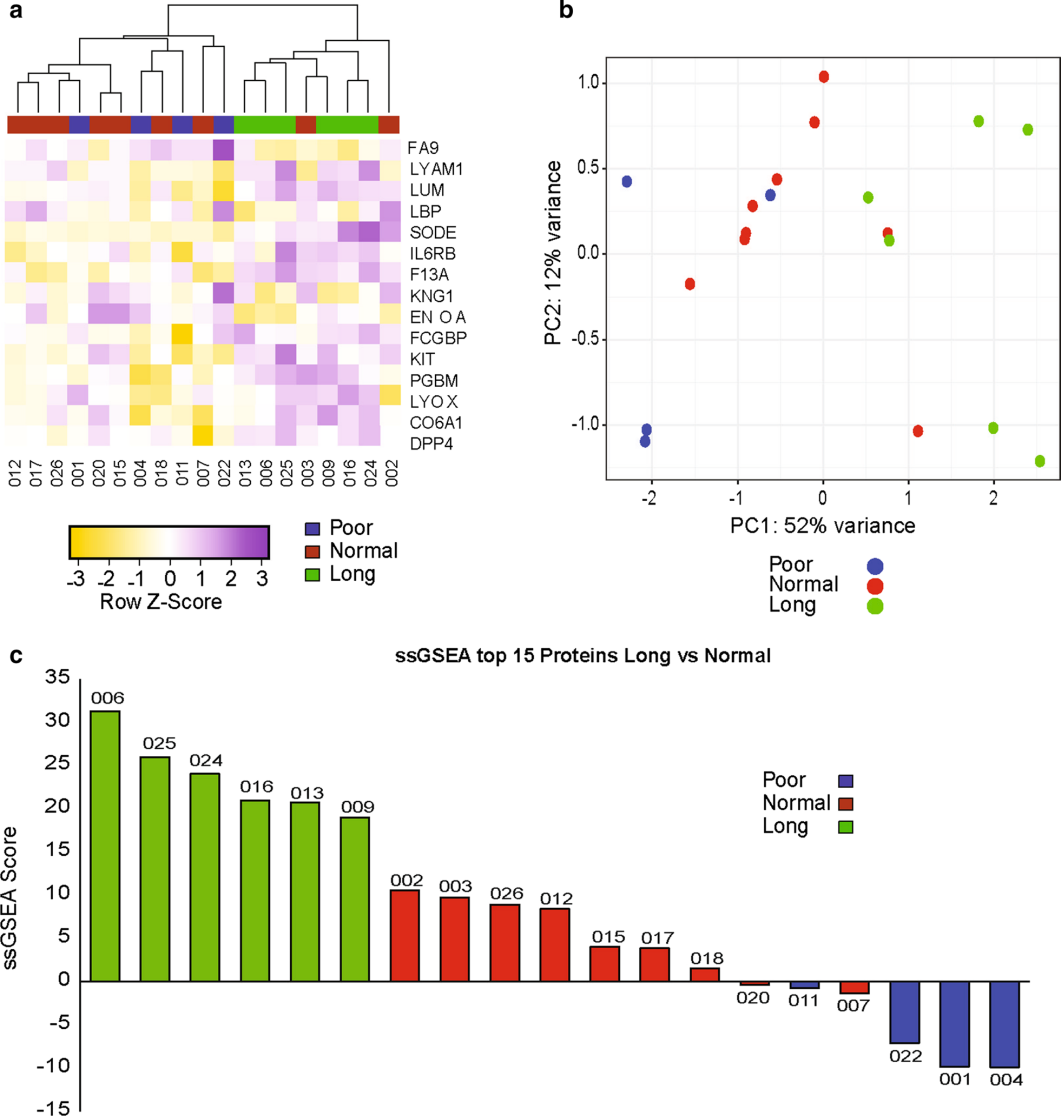
Graphical representation of the top 15 proteins differentially expressed between long and normal responders. Colors represent the duration of response groups, blue for poor responders, red for normal responders and green for long-term responders. **a** Hierarchical clustering of the patients using the 15 proteins differentially expressed between long-term versus normal responders. **b** PCA plot using the same 15 proteins list than (**a**). **c** ssGSEA score was calculated for each patient using the 15 most differentially expressed proteins between long-term and normal responders then ranked

We then used hierarchical clustering and principal component analysis (PCA) to visualize whether these 15 proteins could separate patients according to their response group (Fig. 2a, b). Both methods were successful in separating long-term responders and poor responders. However, the distinction between long-term and normal responders was less obvious. To generate a prognostic score, based on the top 15 proteins, in ALK fusion positive NSCLC patients treated with crizotinib, we applied the single sample Gene Set Enrichment analysis (ssGSEA) method [23], and computed enrichment scores for each patient in our cohort. We found that all patients in the long-term responder group had the highest scores, followed by the normal and then the poor responder groups (Fig. 2c). These preliminary results are encouraging as they demonstrate the presence of a proteomic signal able to distinguish patients with different PFS in response to crizotinib, but unfortunately this method does not allow us to determine which prospective patients will have a longer duration of response to crizotinib.

### Identification of a classifier

Our main goal was to generate a prognostic proteomic signature for NSCLC patients harboring ALK fusion and treated with crizotinib. To identify combinations of proteins that, when taken in concert, could collectively predict patient response duration in the current cohort, an exploratory classifier analysis was performed. We used four machine-learning algorithms [13–15, 17] to better focus the search of likely candidates among the 126 proteins quantitated. Two classifications were performed in parallel: long-term versus normal and long-term versus poor. Proteins that were well-suited to either classification according to the machine-learning algorithms were combined for further analyses (a total of 52 proteins; Additional file 2: Fig. S1b).

In the following step, the optimal panel size that balanced bias and variance was identified by assessing these errors for the panel of increasing size, taking the most important proteins (according to the machine-learning algorithms) first in a a stepwise “greedy” fashion. The optimal panel size was determined to be between 1 and 3 predictors by this method.

Finally, panel analysis was performed using a generalized linear model using the subset of 52 proteins identified by the machine learning algorithms in all possible combinations of 1 to 3 proteins. Numerous panels had an area under the receiver operating characteristic curve (AUC) greater than the pre-selected cut-off of 0.85, especially when comparing long-term versus poor responders (Additional file 4: Fig. S2). Overall, 1914 long-term vs. normal panels and 8377 long-term vs. poor responder panels had AUC greater than 0.85.

### Final Selection of putative proteins for blood-based signature

**Fig. 3:**
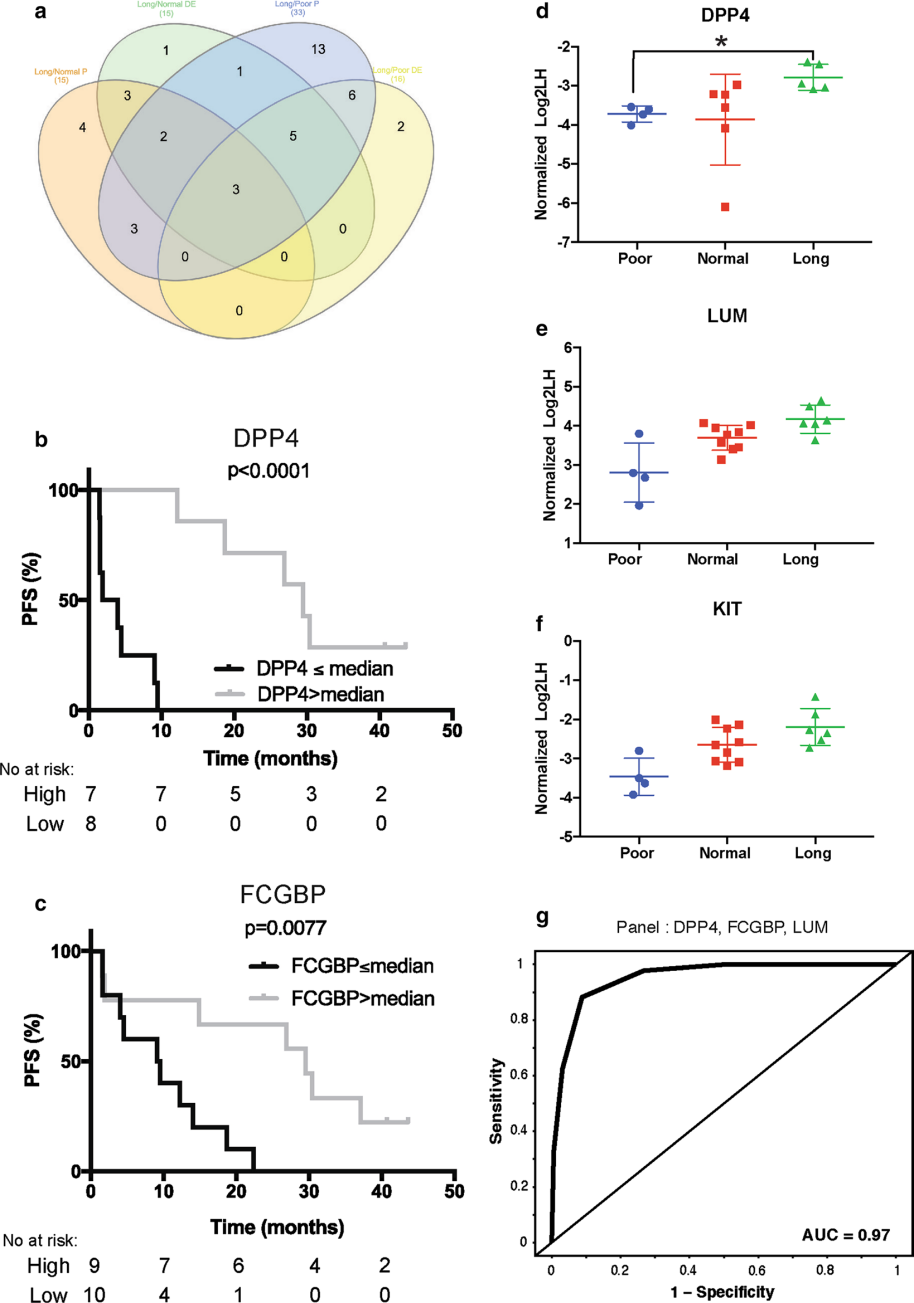
Candidate proteins to classify ALK + NSCLC patients by duration of response to crizotinib. **a** Venn Diagram of the protein list identified with both methods (differential expression = DE, classifier = P) and both comparisons (Long vs Normal and Long vs Poor). Kaplan–Meier plots where patients were separated in two groups based on the median expression value of DPP4 (**b**) or FCGBP (**c**) two of the top contributing protein in long versus normal panel analysis. **d**–**f** Normalized log2LH ratio of three proteins (DPP4, LUM, KIT) in each of the response groups. **g** ROC curve of one of the best panels obtained in long versus normal comparison which includes DPP4, FCGBP and LUM

Considering the huge number of potential panels, we decided to look at the contributions of individual proteins across the panels with AUC greater than 0.85 to identify which proteins should be prioritized. Of the 52 proteins included in the exploratory panel analysis search, 33 (long-term vs. poor) and 15 (long-term vs. normal) were components of more than 5% of the combinations with high performance (AUC greater than 0.85; Fig. 3a and Additional file 2: Fig. S1c). As the number of proteins to prioritize was still high, we decided to intersect the entire list of proteins derived from the various analyses (Fig. 3a), leading to a final list of 22 proteins for putative blood-based signature (Additional file 5: Table S3). Of note, the top protein contributing to the panel in long-term versus normal and long-term versus poor were different, FCGBP and DPP4, respectively; furthermore, each showed a significant relationship between their expression level and PFS (Fig. 3b, c). Interestingly, 3 proteins, DPP4, KIT and LUM, were identified with both methods, making them the most attractive targets. The abundance ratio for each responder group for these three proteins is shown in Fig. 3d–f. Additional file 6: Fig. S3 shows the differential expression of 12 proteins for each responder group. An example receiver operating characteristic curve from a panel of 3 proteins with a promising AUC value (DPP4, FCGBP and LUM) is presented in Fig. 3g.

## Discussion

The use of liquid-biopsy to identify biomarkers for various stages of lung cancer patients has been extensively investigated, with the strongest focus on the diagnosis of malignancy in lung nodules regularly followed by costly repeated radiation exposure to serial CDT imaging [24]. To our knowledge, this is the first report of a blood-based prognostic putative proteomic signature in locally advanced or metastatic ALK fusion positive NSCLC treated with an ALK-TKI.

We used targeted proteomics on blood samples collected from patients prior to crizotinib treatment to identify potential biomarkers of duration of response to ALK tyrosine kinase inhibition. Combining two complementary methods, we identified 22 candidate proteins with prognostic potential in ALK fusion positive NSCLC treated with crizotinib, 3 of which (DPP4, LUM and KIT) were consistently identified in all comparisons and analyses performed. Further validation will be needed using an independent cohort however we believed that this list of proteins is a good starting point for more in depth investigation. Interestingly, previous studies have reported a relationship between the expression of some of these proteins and disease progression.

The first of the top three we identified is LUM (lumican), a glycoprotein which is involved in the extracellular matrix (ECM) formation and regulation and which can have a strong impact on the tumor microenvironnement or stroma function. Modulation of tumor stroma activity can affect apoptotic signaling pathway, facilitate tumor cell migration, angiogenesis, hypoxia and drug delivery all of which are key processes associated with tumor response to treatment. Several previous studies have investigated the role of lumican in tumor biology, for example downregulation of lumican was shown to accelerate lung cancer cell invasion through the p120 catenin pathway [25] and in stage II and III colon cancer patients high expression of lumican in tumor tissues was associated with good clinical outcome [26]. Here, we observed that the plasma level of lumican is also associated with a better prognosis in ALK fusion positive NSCLC patients treated with crizotinib. The second best candidate is CD26/DDP4 a transmembrane glycoprotein with proteolytic activity, which also exists in an enzymatically active soluble form which has been proposed as an important tumor biomarker in different types of cancer [27]. Higher plasma level of DPP4 has been found to be associated to better survival in multiple cancer types combined [28]. Furthermore, soluble low DPP4 level has been suggested to be a prognostic biomarker for colorectal and prostate cancers as well as NSCLC malignant pleural effusions [29–31]. The last candidate is c-KIT transmembrane receptor tyrosine kinase in soluble form. A previous study reported that higher level of soluble KIT in plasma was shown to be associated with enhanced survival in response to sorafenib (another TKI) treatment in advanced hepatocellular carcinoma [32]. Despite the numerous studies showing prognostic values of these three proteins little is known about the biological reasons behind their association with survival and disease progression.

Lung cancer patients are a really challenging population in which to collect high quality tissue specimens and this has led to an increased interest in developing assay and biomarker detection from blood samples. Access to plasma samples from ALK fusion positive NSCLC patients untreated with an ALK-TKI, with associated outcome data following drug administration, is a challenge and that made access to a validation cohort impossible, which is of course one of the main limitations of this study. However, we hope that this discovery work will be a starting point for further studies and collaboration aimed at validating and refining the prognostic protein signature.

As sequential therapy approaches in ALK fusion positive NSCLC patients remain controversial and not yet well defined, our results may provide further insights in clinical decision making about the optimal order of administration of the various ALK-TKI therapies available. Following independent validation, we believe this signature could become a cornerstone in ALK-TKI treatment which may improve the clinical impact of first-line and sequential treatment in ALK fusion positive NSCLC.

## Conclusion

In the present study, we highlighted 22 proteins with prognostic potential in NSCLC patients harboring ALK fusion and treated with crizotinib, alone or in combination under the form of a signature. As resistance remains a major challenge in the treatment of these patients, we believe that developing a signature or biomarkers able to classify patients by duration of response to treatment could lead to better use of the various drugs available to them.

## Supplementary information

**Additional file 1: Table S1.** Patient Groups based on PFS.

**Additional file 2: Fig. S1.** Flow-chart for protein signature discovery. Description of the workflow used to obtain the 22-protein signature. Starting with 327 proteins, only 126 were detected in the majority of the samples. Two methods (a) differential expression and (b) classifier building were applied on the detected proteins leading to the selection of 22 proteins designated as protein signature (c).

**Additional file 3: Table S2.** Top 15 proteins differentially detected between long-term and normal responders.

**Additional file 4: Fig. S2.** Distribution of AUC as a function of panel size. (A) Distribution for the long-term versus normal groups comparison. (B) Distribution for the long-term versus poor groups comparison.

**Additional file 5: Table S3.** Candidate proteins signature for long-term response.

**Additional file 6: Fig. S3.** Expression of all proteins in the signature by duration of response group. Normalized log2LH ratio of all the proteins in the signature separated by response groups. Blue for poor responders, red for normal responders and green for long-term responders.

**Additional file 7: Table S4.** Normalized log2LH ratio of all the proteins measured in all baseline samples.

## Data Availability

All the data used for analysis presented in this manuscript can be found in Additional file 7: Table S4.
