# Supplementary material for: Discovery of a putative blood-based protein signature associated with response to ALK tyrosine kinase inhibition
Source: Clin Proteomics. 2020 Feb 7;17:5. doi: 10.1186/s12014-020-9269-6 (PMC7006423; doi:10.1186/s12014-020-9269-6)
Supplement: Supplementary file 1 — Additional file 1: Table S1. Patient Groups based on PFS. [file 12014_2020_9269_MOESM1_ESM.docx]

| **Additional file 1: Table 1. Patient Groups based on PFS** | |
| --- | --- |
| **Patient ID** | **PFS (months)** |
| **Poor Responders** |  |
| 023 | 1.1 |
| 022 | 1.5 |
| 011 | 1.6 |
| 004 | 1.6 |
| 001 | 1.9 |
| **Normal Responders** |  |
| 003 | 4 |
| 007 | 4.5 |
| 012 | 9.1 |
| 026 | 9.5 |
| 027 | 11.1 |
| 020 | 12.2 |
| 017 | 14 |
| 002 | 14.9 |
| 018 | 18.7 |
| 015 | 22.4 |
| **Long-term Responders** |  |
| 013 | 26.9 |
| 016 | 29.5 |
| 006 | 30.4 |
| 008 | 31.3 |
| 009 | 37.1 |
| 025 | 40.7^1^ |
| 024 | 43.6^1^ |
|  |  |
| ^1^Patient currently on study | |
