# Supplementary figures and images for: Discovery of a putative blood-based protein signature associated with response to ALK tyrosine kinase inhibition
Source: Clin Proteomics. 2020 Feb 7;17:5. doi: 10.1186/s12014-020-9269-6 (PMC7006423; doi:10.1186/s12014-020-9269-6)

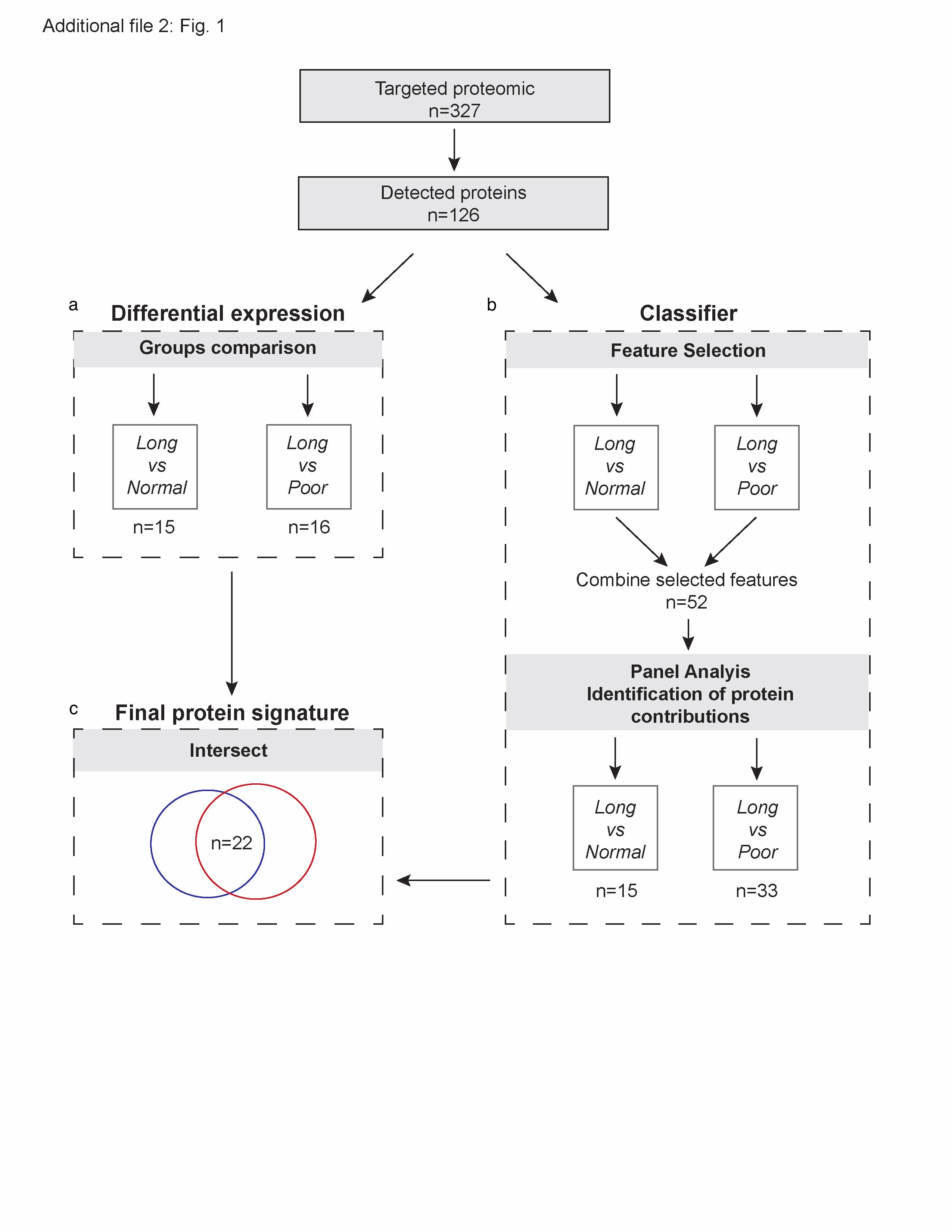

Supplement: Supplementary file 2 — Additional file 2: Fig. S1. Flow-chart for protein signature discovery. Description of the workflow used to obtain the 22-protein signature. Starting with 327 proteins, only 126 were detected in the majority of the samples. Two methods (a) differential expression and (b) classifier building were applied on the detected proteins leading to the selection of 22 proteins designated as protein signature (c). [file 12014_2020_9269_MOESM2_ESM.jpg]

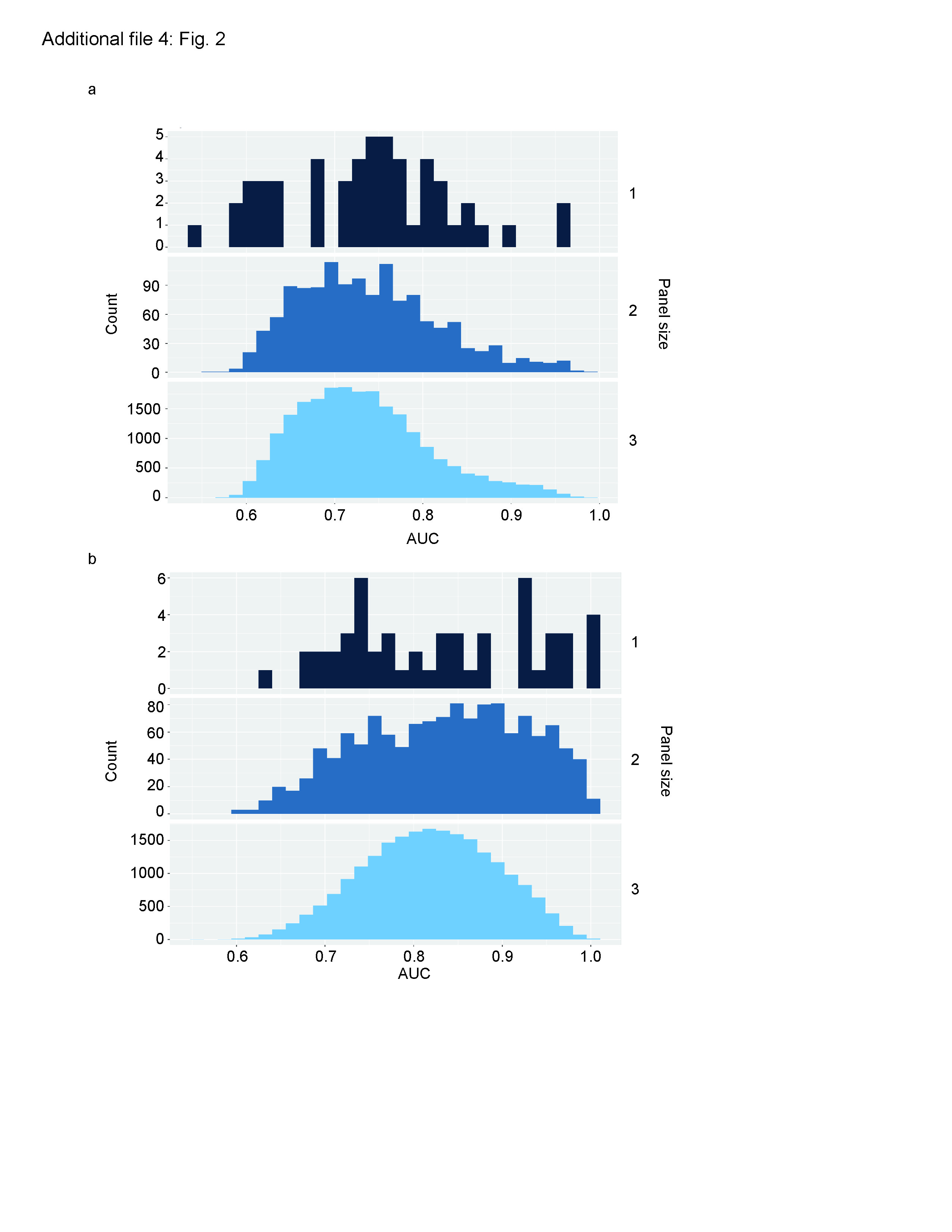

Supplement: Supplementary file 4 — Additional file 4: Fig. S2. Distribution of AUC as a function of panel size. (A) Distribution for the long-term versus normal groups comparison. (B) Distribution for the long-term versus poor groups comparison. [file 12014_2020_9269_MOESM4_ESM.jpg]

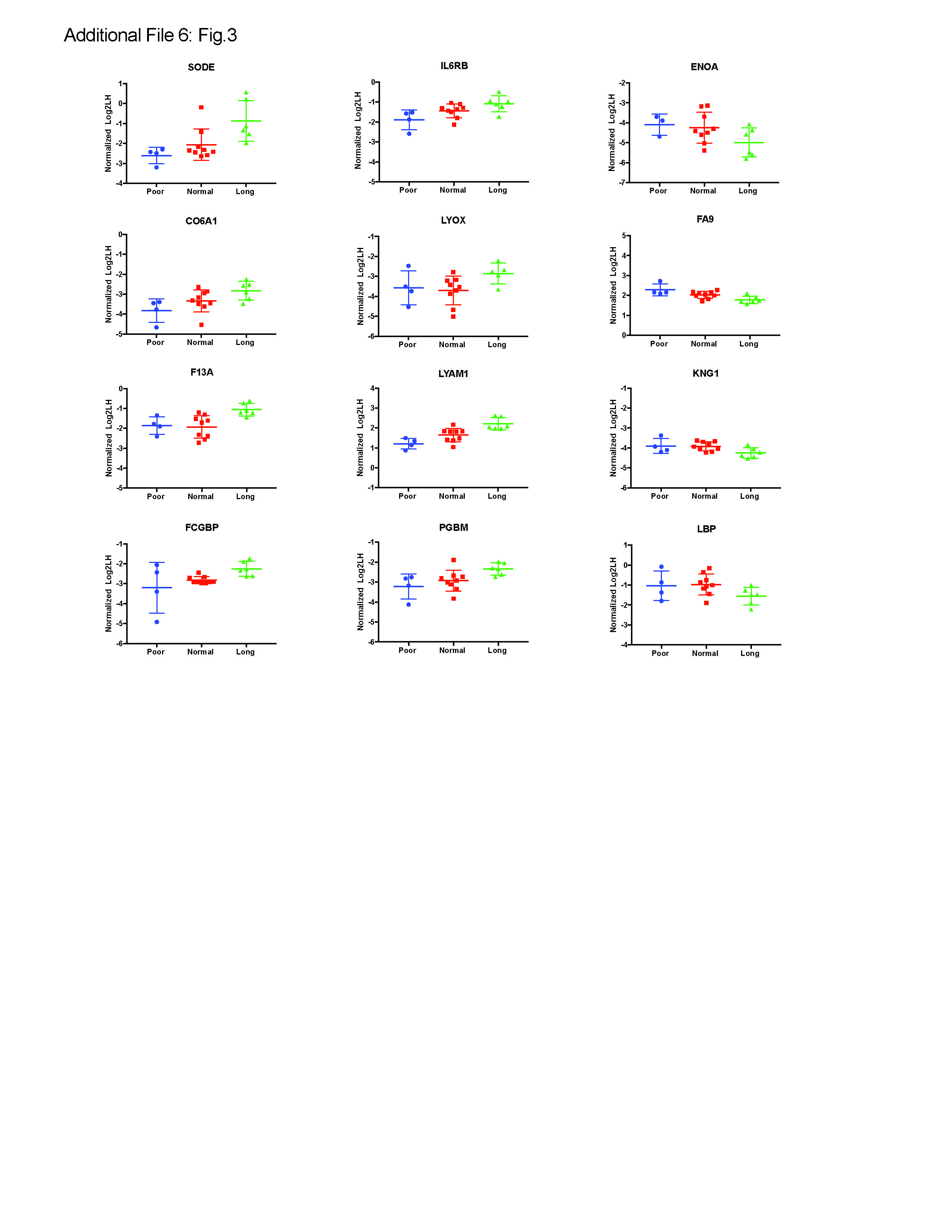

Supplement: Supplementary file 6 — Additional file 6: Fig. S3. Expression of all proteins in the signature by duration of response group. Normalized log2LH ratio of all the proteins in the signature separated by response groups. Blue for poor responders, red for normal responders and green for long-term responders. [file 12014_2020_9269_MOESM6_ESM.jpg]
