# Supplementary material for: Discovery of a putative blood-based protein signature associated with response to ALK tyrosine kinase inhibition
Source: Clin Proteomics. 2020 Feb 7;17:5. doi: 10.1186/s12014-020-9269-6 (PMC7006423; doi:10.1186/s12014-020-9269-6)
Supplement: Supplementary file 3 — Additional file 3: Table S2. Top 15 proteins differentially detected between long-term and normal responders. [file 12014_2020_9269_MOESM3_ESM.docx]

**Additional file 3: Table 2. Top 15 proteins differentially detected between long-term and normal responders.**

|  | | |  |  |  |
| --- | --- | --- | --- | --- | --- |
|  | **Log2 Fold-Change abundance** | | | ***P Value*** |  |
| **Protein** | **Normal Vs Long** | **Poor Vs Long** | | **Normal Vs Long** |  |
| SODE | 1.19 | 1.74 | | 0.013 |  |
| DPP4 | 1.08 | 0.94 | | 0.077 |  |
| F13A | 0.87 | 0.80 | | 0.005 |  |
| LYOX | 0.85 | 0.71 | | 0.038 |  |
| PGBM | 0.59 | 0.88 | | 0.028 |  |
| FCGBP | 0.57 | 0.95 | | 0.001 |  |
| LYAM1 | 0.57 | 1.00 | | 0.005 |  |
| CO6A1 | 0.51 | 0.99 | | 0.086 |  |
| LUM | 0.48 | 1.37 | | 0.016 |  |
| KIT | 0.45 | 1.27 | | 0.080 |  |
| IL6RB | 0.36 | 0.81 | | 0.087 |  |
| FA9 | -0.25 | -0.50 | | 0.020 |  |
| KNG1 | -0.33 | -0.35 | | 0.020 |  |
| LBP | -0.60 | -0.54 | | 0.039 |  |
| ENOA | -0.74 | -0.90 | | 0.085 |  |
