# Supplementary material for: Discovery of a putative blood-based protein signature associated with response to ALK tyrosine kinase inhibition
Source: Clin Proteomics. 2020 Feb 7;17:5. doi: 10.1186/s12014-020-9269-6 (PMC7006423; doi:10.1186/s12014-020-9269-6)
Supplement: Supplementary file 5 — Additional file 5: Table S3. Candidate proteins signature for long-term response. [file 12014_2020_9269_MOESM5_ESM.docx]

**Additional file 5: Table 3. Candidate proteins signature for long-term response.**

| **Protein** |  |
| --- | --- |
| **DPP4 ^a^** |  |
| **KIT ^a^** |  |
| **LUM ^a^** |  |
| FCGBP |  |
| F13A |  |
| ENOA |  |
| TCO1 |  |
| LYAM1 |  |
| CO6A1 |  |
| IL6RB |  |
| PGBM |  |
| SODE |  |
| FRIL |  |
| MMP9 |  |
| TSP1 |  |
| C1QR1 |  |
| CO1A2 |  |
| ANGT |  |
| KNG1 |  |
| LYOX |  |
| FA9 |  |
| LBP |  |

^a^ identify in all type of analysis.
